# Variation in Immune Parameters and Disease Prevalence among Lesser Black-Backed Gulls (*Larus fuscus* sp.) with Different Migratory Strategies

**DOI:** 10.1371/journal.pone.0118279

**Published:** 2015-02-13

**Authors:** Elena Arriero, Inge Müller, Risto Juvaste, Francisco Javier Martínez, Albert Bertolero

**Affiliations:** 1 Department of Migration and Immunoecology, Max Planck Institute for Ornithology, Radolfzell,Germany; 2 Department of Zoology and Anthropology, University Complutense of Madrid, Madrid, Spain; 3 Department of Biology, University of Konstanz, Konstanz, Germany; 4 Department of Biology, University of Turku, Turun yliopisto, Finland; 5 Department of Microbiology and Parasitology, University of Alcala, Alcalá de Henares, Spain; 6 Institute of Aquatic Ecosystems (IRTA), Sant Carles de la Ràpita, Spain; Pennsylvania State University, UNITED STATES

## Abstract

The ability to control infections is a key trait for migrants that must be balanced against other costly features of the migratory life. In this study we explored the links between migration and disease ecology by examining natural variation in parasite exposure and immunity in several populations of Lesser Black-backed Gulls (*Larus fuscus*) with different migratory strategies. We found higher activity of natural antibodies in long distance migrants from the nominate subspecies *L*.*f.fuscus*. Circulating levels of IgY showed large variation at the population level, while immune parameters associated with antimicrobial activity showed extensive variation at the individual level irrespective of population or migratory strategy. Pathogen prevalence showed large geographical variation. However, the seroprevalence of one of the gull-specific subtypes of avian influenza (H16) was associated to the migratory strategy, with lower prevalence among the long-distance migrants, suggesting that migration may play a role in disease dynamics of certain pathogens at the population level.

## Introduction

Migration has always attracted much attention as a fascinating life strategy involving displacements which can span several continents [1]. At the same time, migration still poses many questions regarding the considerable energetic demands and sophisticated physiological adaptations and mechanisms of navigation [1–3]. More recently, migration has attracted attention as an important factor presumably involved in spreading zoonotic diseases [4].

Migrants are exposed to a wide variety of environments, including all their biotic and abiotic components. Pathogens are an important biotic component of the environment and represent one of the major challenges for migrants [5,6]. Several phenomena imply that migratory animals may suffer higher impact from parasitism than non-migrants. Those include higher encounter rate with parasites from different areas (e.g. [7,8]), immunomodulation due to physiological demands incurred in migration [9,10], relapse of chronic infections prior or during the trip [11,12], or increased risk of infection due to host aggregation during migration or at the wintering sites [13].

However, despite the exposure to a broader range of pathogens and high susceptibility to infection presumably associated with long-distance migration, at the population level, the prevalence of infection may be lower in migrants than in residents, and some recent studies advocate for a role of migration in keeping populations healthy [14]. Several non-excluding hypotheses have been proposed to explain the role of migration in disease ecology. On one hand, from the perspective of the selection pressure imposed by parasitism, animals are expected to match their investment in immune defence to the parasitic threats they face, and in that context, migrant birds are expected to invest highly in immune defense [14,16]. Under this scenario, we would expect to find differences in parasite prevalence associated with migratory strategy and also associations between mechanisms of disease resistance and migration. At the interspecific level, a comparative study across 263 bird species showed lower prevalence of hemosporidian parasites in migrant species than in residents, although no explicit association between immunity and migration was tested in that study [18]. On the other hand, lower parasite prevalence in migrants has been proposed to be mediated by mechanisms such as migratory escape from infected areas [19], or migratory culling of sick individuals due to the exacerbated physiological demands of the trip and the infection [6]. Evidences from parasite prevalence in monarch butterflies support this role of migration in host-parasite dynamics [16,20]. This alternative scenario does not entail predictions about different immunity in migrants and residents. Thus, the main two mechanisms proposed underlying associations between migration and disease dynamics are elimination of infected individuals from the population, or selection for higher investment in mechanisms of disease resistance.

In the last decades, several animal species have shown changes in their migratory habits [21]. Changes in migratory strategy may be followed by changes in physiological adaptations and different host-pathogen dynamics, which may result in higher or lower disease risk [14,17]. In migratory birds with wide-ranging dietary preferences, the cost-benefit balance is shifted towards resident life when food is available year round in human-made settings like rubbish dumps [1]. Migration imposes tremendous physiological costs that may drive numerous trade-offs and physiological adaptations, including the investment in immune defence [6]. Therefore, changes towards a resident life style may alter for example migration-mediated immunomodulation [17,22].

On the other hand, variation in migratory strategy can result from the selection pressures that lead to species divergence [23]. When a taxonomic group diversifies in different migratory strategies, phenotypic or behavioural changes may be followed by changes in physiological adaptations [24].

The aim of this study is to examine variation in immune parameters and exposure to pathogens in a taxonomic group exhibiting wide variation in migratory strategies to test for associations between migration and parasite prevalence and immunity. The Lesser Black-backed Gull is a suitable model, because the taxonomic group includes several subspecies with different migratory habits, and gulls are a good example of recent changes in migratory behavior driven by human environmental modification. The Lesser Black-backed gull group (*Larus fuscus*) comprises three subspecies *L*.*f*. *fuscus*, *L*.*f*. *intermedius* and *L*.*f*. *graellsii* [25,26]. Although the subspecies grade into each other and have no clear limits, taxon designation is based on geographic location of the breeding colony and phenotypic differences [26]. The subspecies of Lesser Black-backed Gulls differ in dorsal plumage colour, ranging from black in the nominate taxa (from Northern Europe) to slate-grey in *graellsii* (from South-western Europe) [27]. The migratory strategy also varies within the Lesser Black-backed group: the nominate *L*.*f*. *fuscus* migrates long distances mainly to tropical Africa where it overwinters in large inland lakes, while *intermedius* and *graellsii* adopted a more resident life strategy and are medium or short distance migrants, wintering in South-western Europe and North-western Africa [1]. Despite the marked phenotypic differences in plumage color and migratory habits, molecular analyses show little genetic variation and poor phylogeographical structure within the Lesser Black-backed group [25].

## Material and Methods

### Study populations and sampling

**Fig 1:**
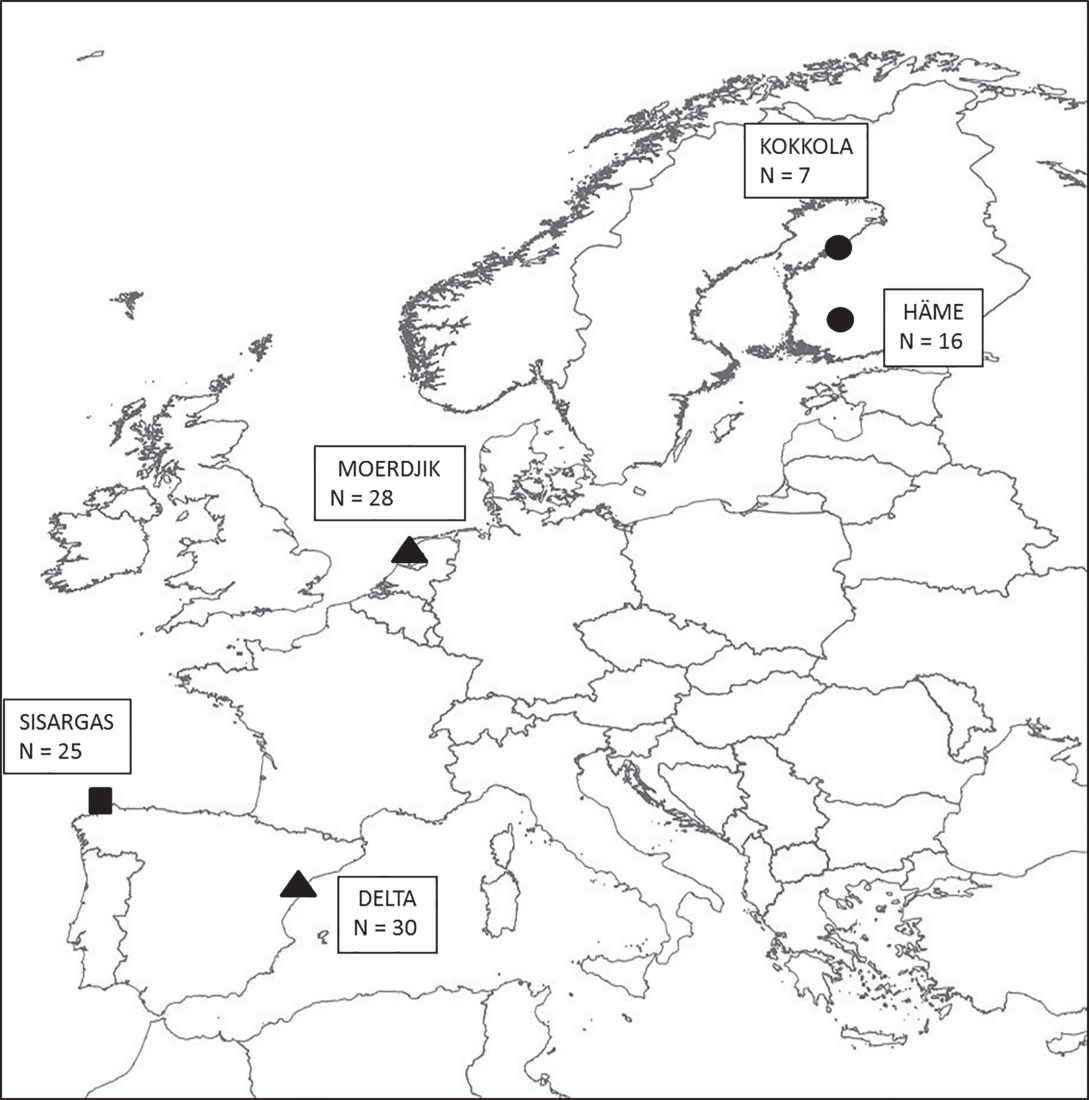
Location of the breeding populations and number of individuals sampled. (Sisargas (Galicia, Spain); Delta (Ebro delta, Spain); Moerdjik (The Netherlands); Häme (Finland); Kokkola (Finland). Häme and Kokkola are long-distance migrants. Different symbols denote subspecies: circles *L f*.*fuscus*, triangles *Lf*. *Intermedius*, squares *Lf*.*graellsii*

The study was conducted during the breeding season of 2009 in five localities distributed along Europe, and including the only breeding colony in the Mediterranean. The five sampling sites include the subspecies *graellsii* (population from Northwest Spain (Sisargas)), *intermedius* (populations from the Netherlands (Moerdjik) and Northeast Spain (Ebro delta)), and *fuscus* (populations from Finland, Häme and Kokkola) (Fig. 1). Populations from Finland (and in general from Scandinavia) are considered long-distant migrants, as their main wintering sites are located in lakes from East Africa [28]. Lesser black-backed gulls from The Netherlands have been reported to winter predominantly along the coasts of South-western Europe, and are considered short-distance migrants [29]. Although there are, to our knowledge, no published data about the migratory strategy of the two colonies from the Iberian peninsula (colonies from Sisargas and Ebro Delta), ringing data, and especially observations of color rings outside the breeding season provide support for a short-distance migratory strategy in these colonies (20 out of the 25 gulls ringed in Sisargas for this study have been reported at least once wintering in the Mediterranean coast or Atlantic coast, and ringing data from Ebro delta showed that the Mediterranean area is the main wintering area for individuals of that colony, with only 6.3% of birds reported wintering in the Atlantic coast. In each locality ca. 25 adult breeding birds were captured at the nest with walk-in traps and blood samples and oropharyngeal and cloacal swabs were obtained according to standard sampling procedures. Heparinized whole blood samples were obtained from the wing vein (Vena ulnaris) and centrifuged (1000 G, 10 min) in order to obtain blood plasma. Plasma samples were stored at -20°C until analyses were performed. Swab samples (Virocult, Medical Wire and Equipment Co Ltd, Corsham, UK) were stored and shipped at 4°C and arrived in the laboratory within less than three days after sampling. All samples were obtained thanks to collaboration with local groups that were already monitoring breeding populations and catching adult birds. Procedures were approved by Finnish National Animal Experiment Board (ESLH-2009-03944/Ym-23). Sampling in the different locations was approved by local and regional authorities; Galicia: Dirección Xeral de Conservación da Naturaleza (Xunta de Galicia), Ebro Delta: Servei de Protecció i Gestió de la Fauna (Generalitat de Catalunya), The Netherlands: Vogeltrekstation, Finland: National Animal Experiment Board. Researchers involved in the design and sampling are certified in the use of animals for research purposes according to the current European legislation.

### Immune parameters

Natural antibodies and complement activity

The activity of natural antibodies and complement proteins were used as an estimation of constitutive immune defenses. Natural antibodies have been classified as constitutive components of both the innate and adaptive immune defense [30,31], while the activity of complement proteins is considered a constitutive innate defense [32]. Natural antibodies and complement cascade provide a first line of defense against pathogens. We estimated the activity of both components through the hemolysis-agglutination assay developed by Matson et al.[31]. In brief, plasma samples were serially diluted twofold with 0.01M phosphate buffered saline (PBS Sigma P3813) in 96-well round bottom plates (Corning Costar #3795) and incubated at 37°C for 90 min with a 1% rabbit blood cell suspension. A negative control of PBS was added in all the plates. Natural antibody (Nab) titers and complement activity were scored as –log_2_ of the highest dilution exhibiting agglutination (Nab) or hemolysis (complement).

Total IgY

Immunoglobulin levels were determined by a direct enzyme-linked immunosorbent assay (ELISA) optimized for this study following Martinez *et al*. [33]. Plates were coated with plasma diluted 1:4000 in 0.1M carbonate-bicarbonate buffer. A peroxidase-conjugated polyclonal rabbit anti-chicken IgY (Sigma A-9046) was used and colour reactions were developed by incubation with the substrate ABTS (Sigma A-1888). Absorbance was measured at 405nm with a plate reader (VersaMax ELISA Microplate Reader, Molecular Devices, Inc., Sunnyvale, CA, USA).

Haptoglobin concentration

Haptoglobin is an acute phase protein involved in binding free *haem* released into the circulation [34]. Haptoglobins play an important role as immunomodulators, and plasma circulating levels may increase several fold in the face of inflammatory stimulus, such as those caused by infections [34]. To estimate haptoglobin concentration in plasma we measured pseudoperoxidase activity of haemoglobin, which is directly proportional to the concentration of haptoglobin in the sample. We used a commercial haptoglobin test kit (TP-801, Tridelta Development Limited, Maynooth, Co. Kildare, Ireland), and plates were read at 630nm. OD values were ln-transformed before the analyses.

Lysozyme concentration

Plasma lysozyme levels were estimated following the assay adapted by Millet *et al*. [35]. We prepared a suspension of 50ml 1% agar (Difco, Detroit, MI) with 25mg dried *Micrococcus lysodeikticus* (Sigma M3770). Then 300μl of this suspension and 15μl of plasma sample from the birds were added in duplicates to a 96-well plate. We obtained a standard curve of lysozyme concentration by adding the bacterial suspension to serial dilutions of a standard lysozyme (Sigma L-6876) solution. Plates were incubated overnight at room temperature and absorbance was measured after 18hrs at 650nm with a microplate reader. Results were analyzed in ln-transformed OD units. Higher OD values indicate lower lysozyme activity.

Leucocyte profile

Leucocyte profile provides information on circulating immune cells and can be used as an indicator of current infections [36]. Relative numbers of the different leucocyte types were obtained examining blood smears stained with Giemsa under a light microscope at 1000x magnification. We obtained relative numbers of the different leucocyte types (heterophils, eosinophils, basophils, lymphocytes, monocytes) up to a total of 100 leucocytes. We calculated the H/L ratio as an indirect index of physiological stress [37].

### Pathogen prevalence

Infections by blood parasites from the genus *Plasmodium* or *Haemoproteus* were assessed by amplifying a fraction (390bp) of the cytochrome-b gene of the parasite, using the primer pair Palu-F/Palu-R [38]. PCR products were visualized in 1.5% agarose gel and amplicons of positive samples were sequenced. Infections were confirmed by light microscopy with blood smears stained with Giemsa.

The infection status of viral infections was determined using swab and serum samples. Oropharyngeal and cloacal swabs were tested for avian influenza by real-time RT-PCR targeting the viral M-gene (Super-ScriptIII One-Step RT-PCR Kit with Platinum Taq polymerase, Invitrogen). To detect prior infections with avian influenza viruses (AIV) and avian paramyxoviruses (APMV) serum samples were analyzed by ELISA and hemagglutination inhibition assays (HI-test). Antibodies directed against the AIV nucleocapsid protein were detected using a competitive NP-ELISA (cELISA; ID Screen, Influenza A NP-Antibody Competition; ID.VET, Montpellier, France). The results were evaluated as percent inhibition (PI = 1 - (OD sample/mean OD negative) x 100). Samples with a PI <46% were classified as positive, samples with a PI>46% were classified as negative [39]. Positive samples were subsequently analysed by hemagglutination inhibition assays (HI-test) following standard procedures [39], and using a panel of four different reference antigens including the subtypes H5, H7, H13 and H16. All serum samples were additionally tested by HI-test for antibodies against three avian paramyxovirus serotypes APMV-1/Clone 30, APMV-4/duck/HongKong/D3/75 and APMV-6/duck/HongKong/199/77, following standard procedures [40].

### Statistical analyses

Variation in immunological parameters was analyzed using linear mixed effects models, with the different immunological parameters as the dependent variable, and sex and infection status as fixed factors, and population, subspecies and migratory strategy as random factors with hierarchical structure (populations within subspecies and subspecies within migratory strategy). Models were subsequently simplified based on AIC values and likelihood ratio test of model comparison. Because several studies have shown sexual differences in immune parameters, [41], in the prevalence of Haemoproteus infections [42], or in the immune response to viral infections [43], sex was included in all statistical models as a fixed effect, and subsequently removed when the fit of the model was not improved. Variation in pathogen prevalence was analyzed with generalized linear mixed effects models with the infectious status (1/0) as the dependent variable with binomial distribution and the same hierarchical structure of random factors. A total of 106 samples were available for this study (48 males, 58 females), although not all the assays could be performed with all the samples due to technical issues, and therefore the sample size slightly varies in the statistical analyses. Means ± standard deviations are reported through the text. Analyses were performed using R 3.1.1 (http://www.R-project.org).

## Results

### Immune parameters

**Fig 2:**
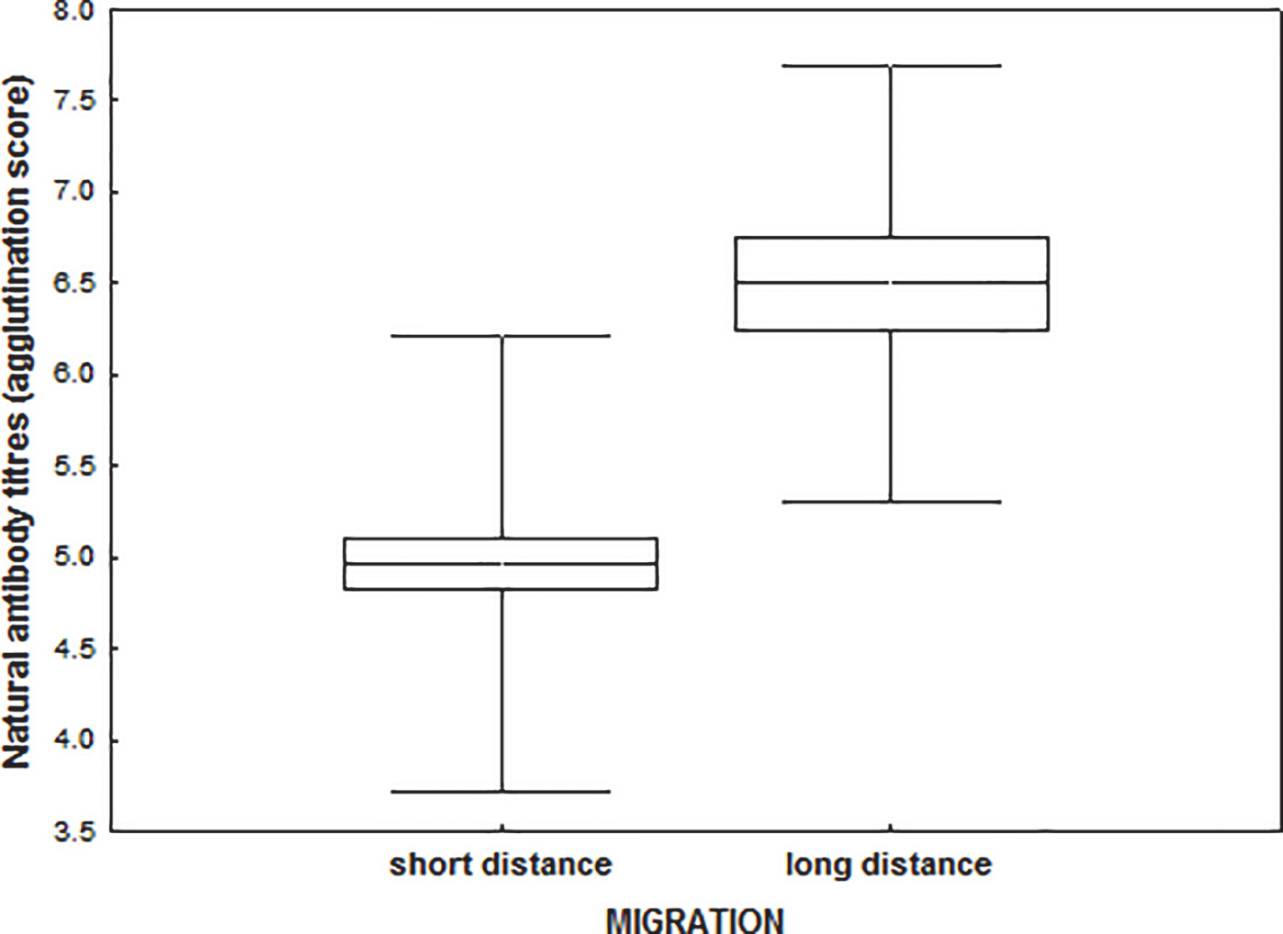
Natural antibody titres in short- and long-distance migrants. Mean, SE and SD.

Migratory strategy explained 33% of the variation in the concentration of natural antibodies, with long distance migrants showing higher titers than short distance migrants (agglutination score long distance: 6.50 ± 1.20, short distance: 4.96±1.25, Fig. 2). Population identity explained 9% of the variation in natural antibodies, while subspecies explained less than 1% of the variation (Table 1). The best-fit model also included prevalence of *Haemoproteus* as a significant explanatory variable of individual variation in natural antibodies, with infected birds showing lower levels than uninfected birds (*t* = -3.181, *P* = 0.002, non-infected: 5.66 ± 1.35, infected: 4.61±1.19). Total IgY levels showed significant variation among populations (19% of variance explained), but migratory strategy explained less than 1% of the variance (Table 1). The model included sex (*t* = 1.752, *P* = 0.083), *Haemoproteus* prevalence (*t* = 3.247, *P* = 0.002) and seroprevalence of avian influenza (*t* = 2.312, *P* = 0.023) as explanatory variables, with males and birds which tested positive for the parasitic infection showing higher IgY concentrations. We also found a significant interaction between sex and prevalence of *Haemoproteus* (*t* = -2.126, *P* = 0.036), showing that in females, but not in males there was a significant increase in IgY levels associated with infection. The interaction between avian influenza and *Haemoproteus* prevalence was also significant (*t* = -3.622, *P* < 0.001), showing a much higher increase in IgY levels when infected by haemoparasites among birds which showed no previous exposure to avian influenza.

**Table 1:** Mean ±SD for immunological parameters in the five locations of the study.

	Short-distance migrants			Long distance migrants	
Immune parameter	Sisargas	Delta	Moerdjik	Häme	Kokkola
Natural antibodies	4.52 ± 1.00^a^	5.50 ± 1.46^b^	4.76 ± 0.97^ab^	6.78 ± 1.28^c^	5.86 ± 0.69^abc^
Total IgY (OD)	2.80 ± 0.58^bc^	2.36 ± 0.50^a^	2.59 ± 0.60^ab^	3.18 ± 0.25^c^	2.62 ± 0.51^abc^
Haptoglobin (OD)	0.10 ± 0.11^ab^	0.08 ± 0.05^a^	0.18 ± 0.17^b^	0.09 ± 0.14^ab^	0.12 ± 0.09^ab^
Complement activity	3.20 ± 0.46	3.07 ± 0.49	2.78 ± 0.56	3.50 ± 1.86	2.57 ± 1.13
Lysozyme activity (OD)	1.11 ± 0.18	1.03 ± 0.05	1.03 ± 0.11	1.06 ± 0.09	1.02 ± 0.07
Heterophils (%)	44.55 ± 18.37	40.67 ± 18.28	35.97 ± 14.26	41.63 ± 12.92	45.40 ± 13.82
Lymphocytes (%)	54.04 ± 18.37	58.78 ± 18.00	63.11 ± 14.35	57.66 ± 12.88	53.60 ± 13.27
H/L ratio	1.06 ± 0.80	0.89 ± 0.75	0.67 ± 0.50	0.84 ± 0.60	0.96 ± 0.56

Short-distance migrants: (Sisargas (Galicia, Spain); Delta (Ebro delta, Spain); Moerdjik (The Netherlands); Long-distance migrants: Häme (Finland); Kokkola (Finland). Units: natural antibodies (-log_2_ agglutination score), complement (-log_2_ lysis score), IgY, Haptoglobin and Lysozyme (Optical density). Higher OD values indicate lower lysozyme.

^abc^ denote differences among populations in post-hoc statistical analyses.

Immune parameters mainly correlated with antimicrobial activity such as complement and lysozyme activity did not show significant differences among populations, and migratory strategy explained less than 1% of the variation observed in these parameters (Table 1). We did not find association between pathogen prevalence and complement or lysozyme activity (i.e. none of the variables of pathogen prevalence were retained in the final models). However, lysozyme activity was higher in females than in males (*t* = -2.695, *P* = 0.008; females: 1.08 ± 0.14, males: 1.02 ± 0.07).

Haptoglobin levels were not associated with migratory strategy, but 8% of the variation was explained by differences among populations (Table 1). Birds suffering from *Haemoproteus* sp. infections had lower levels of haptoglobin (*t* = -2.055, *P* = 0.043, infected: 0.08 ± 0.07; non-infected: 0.13 ± 0.14). Leucocyte profiles and H/L ratio did not show any pattern with migratory strategy or subspecies, and we did not find significant variation among populations (Table 1). No association between leucocyte profiles and infectious status was found.

Natural antibody titers were positively and significantly correlated with complement activity (*t* = 5.139, *P* < 0.001). Lysozyme activity was negatively correlated with IgY (t = -2.071, *P* = 0.041).

### Pathogen prevalence

Total prevalence of protozoan blood parasites was 34%. Haemoparasites were identified as belonging to the genus *Haemoproteus*, except for two individuals infected by *Parahaemoproteus*. Variation in Haemoproteus prevalence was explained by a model that included population id, but migratory strategy and subspecies explained less than 1% of the variance and were subsequently removed as explanatory factors (Table 2). The other variables retained in the best-fit model were the activity of natural antibodies and plasma concentrations of haptoglobin, with infected birds showing lower concentration of natural antibodies (*z* = -2.557, *P* = 0.011), and a trend for lower concentration of haptoglobin (*z* = -1.817, *P* = 0.069; see mean values above).

**Table 2:** Pathogen prevalence (current protozoan infections and viral serology, see methods) in the five populations of the study.

	Short-distance migrants			Long-distance migrants	
Prevalence	Sisargas	Delta	Moerdjik	Häme	Kokkola
Haemoproteus	0.56^b^	0.17^a^	0.43^b^	0.31^ab^	0.00^a^
Influenza A	0.88^a^	0.80^a^	0.61^ab^	0.44^b^	0.71^ab^
H5	0.24	0.47	0.17	0.06	0.29
H7	0.04	0.00	0.00	0.00	0.00
H13	0.68	0.77	0.39	0.31	0.43
H16	0.80^a^	0.67^a^	0.57^a^	0.19^b^	0.29^b^
APMV-1 (NDV)	0.38^b^	0.70^a^	0.24^b^	0.38^b^	0.50^ab^
APMV-6	0.08^b^	0.50^a^	0.05^b^	0.44^a^	0.67^a^

(Sisargas (Galicia, Spain); Delta (Ebro delta, Spain); Moerdjik (The Netherlands); Häme (Finland); Kokkola (Finland). Significant differences among populations are denoted by letters.

^ab^ denote differences among populations.

**Fig 3:**
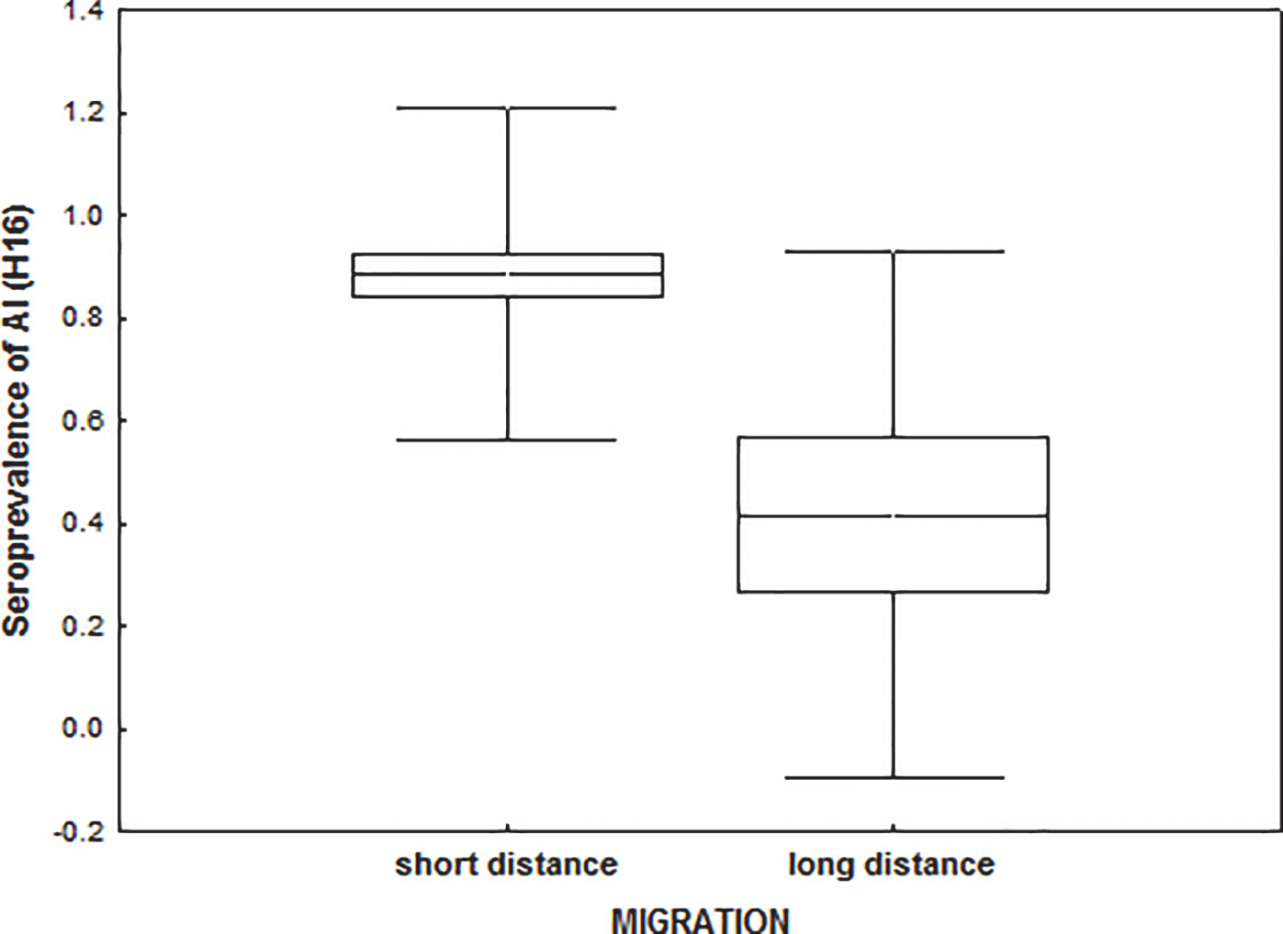
Seroprevalence of Avian influenza—hemagglutinin subtype H16 in long-distance and short-distance migrants. Mean, SE and SD.

Exposure to the different viral infections tested varied greatly among populations (Table 2). All samples were tested negative for the presence of AIV-RNA with RT-PCR; ongoing infections with influenza A viruses at the time of sampling could thus be excluded. Overall seroprevalence of avian influenza (AI) was high (68.3%), with significant variation among populations (*z* = 3.481, *P*< 0.001, Table 2). Migratory strategy and subspecies did not explain differences in seroprevalence and were not retained in the final model. However, we found a significantly lower proportion of males than females with anti-influenza antibodies (z = -2.66, *P* = 0.007, males: 0.54, females: 0.83). H16 was the most common AI-subtype detected, and its prevalence among the AI seropositives was explained by migratory strategy (*z* = 3.009, *P* = 0.003), with long distance migrant populations showing lower prevalence (Table 2, Fig. 3). Seroprevalence of H13 and H5 did not differ significantly among populations, and H7 was only detected in an individual from Islas Sisargas (Spain). Presence of antibodies against several hemagglutinin subtypes in the same individual was relatively common, but we did not find significant differences among populations.

Seroprevalence of Avian paramyxovirus serotypes differed significantly among populations (Table 2). The proportion of individuals with detected antibodies against Newcastle Disease Virus (APMV-1) was 45%, and population id was the only explanatory factor retained in the statistical model (*z* = 1.989, *P* = 0.045). Total seroprevalence of APMV-6 was 30%, also with significant differences among populations (Table 2). The best-fit model for APMV-6 included population id (*z* = -1.743, *P* = 0.081) and plasma levels of IgY (*z* = 1.757, *P* = 0.079) as explanatory variables, with slightly higher IgY levels in birds seropositive to APMV-6 (negative: 2.65, positive: 2.76). Subspecies and migratory strategy explained less than 1% of the variance in seroprevalence of APMV-6 and APMV-1, and were not retained in the final models. We did not detect exposure to APMV-4. In total, 17% of the birds had antibodies against multiple viral strains, but there were no significant differences among populations (*P* = 0.65).

## Discussion

Our study shows significant geographical variation in pathogen prevalence and immune parameters in Lesser Black-backed Gull populations with different migratory strategies. We found higher activity of natural antibodies in gulls of the long-distance migrant nominate subspecies *Larus fuscus fuscus*, suggesting that long-distance migrants maintain higher constitutive immunity than short-distance migrants.

Since the end of the 60’s the Western populations of Lesser Black-backed Gulls adopted an omnivorous diet and a resident life style [1]. Avoiding tropical areas and less energetically demanding migratory habits [29] seem to be associated with lower investment in constitutive antibody-mediated immunity in the subspecies *intermedius* and *graellsii*. Several studies have highlighted the important role of the environment in explaining geographical variation in immune parameters at intra- or interspecific level [44–47].

The taxonomic group constituted by *Larus fuscus* has been reported to have little genetic variation [48]. Our study shows little differentiation in physiological parameters involved in immune defense among subspecies, which suggests a minor role of genetic effects explaining the variation found in immunity and disease susceptibility in our study [49,50]. The different subspecies of *Larus fuscus* have undergone some degree of phenotypic differentiation in morphological characters, (e.g. plumage coloration) and in migratory habits [51]. However, only the nominate subspecies *L*. *fuscus fuscus* exhibits differences in physiological parameters involved in the immune response. This may have important implications in the susceptibility to pathogens and the ability to control infections [52], although not all pathogens had lower prevalence in the Finnish population, and variation in pathogen prevalence was not explained at the taxonomic level of subspecies.

We found large geographical differences in the prevalence of some of the major avian diseases in Lesser Black-backed Gulls. Furthermore, short-distance migratory strategy was associated with higher seroprevalence of one of the gull-specific subtypes of avian influenza, H16. Although we do not have serologic evidence of exposure to highly pathogenic strains of avian influenza virus in the gulls of our study, infections with low pathogenic strains of avian influenza have been reported to impact migratory performance in other species [53,54]. Thus, infections with low pathogenic strains of avian influenza may have higher clinical and ecological consequences than previously thought, or may have exacerbated effects due to other concurrent infections, highlighting migratory culling as a likely explanation for the lower seroprevalence of H16 found in long-distance migrants. Further support for migratory culling is provided by the fact that seroprevalence indicates the proportion of individuals which have been infected and survived an infection by that particular serotype of avian influenza.

Our results show that over 50% of the birds tested had been exposed to different hemagglutinin subtypes of AIV, and a large proportion of them had also been exposed to several avian paramyxovirus serotypes, including New Castle Disease Virus (APMV-1). Prolonged use of habitats may explain high prevalence of pathogens with oral-fecal mode of transmission [55,56]. This may explain the important spatial variation found in our study in exposure to avian viruses, and the remarkably high seroprevalence in some of the colonies tested (Table 2).

Anseriformes (ducks, geese, swans) and Charadriiformes (gulls, terns, shorebirds) are considered the main natural reservoirs for avian influenza viruses, and important vectors of several other infectious diseases (e.g.[57–59]). In particular, gulls are considered key in facilitating intercontinental exchanges of AIVs [60] and may have a relevant epidemiological role because of their close contact with human settlements [61].

Despite the fact that none of the birds tested in our study were shedding influenza viruses at the time of sampling, we found high seroprevalence rates (52–88%), indicating that a large proportion of the gulls had been into contact with the virus and had mounted an immune response [62]. Our results are comparable to previous results obtained in other gull species (e.g. [63,64]), and indicate that the Lesser Black backed Gulls of the five populations sampled have been exposed to several hemagglutinin subtypes, with H13 and H16 being the most prevalent. H13 and H16 are considered gull-specific subtypes [65–67], and their maintenance in gull populations is attributed to high incidence of LPAIV among first year birds [68]. In our study, adult gulls were sampled in the early breeding season (i.e. before hatching), suggesting that the high seroprevalence of H13 and H16 is an indication of perpetuation of these serotypes within the populations.

Interestingly, Lesser Black backed Gulls had antibodies against the hemagglutinin subtypes H5 and H7, which are potential precursors of highly pathogenic avian influenza and are considered notifiable diseases with special regulatory measures in most countries. However, while individuals from all colonies sampled were seropositive for H5 (with seroprevalence ranging between 13–47%), only a single individual from the colony in North-Western Spain tested positive for antibodies against H7.

We found an overall seroprevalence of APMV-1 of 45%. Although velogenic strains of APVM-1 (NDV) are the causation agent of Newcastle Disease, one of the most devastating diseases of poultry, the strains of the virus commonly found in waterfowl and sea birds are considered avirulent [55]. These strains grow mainly in the intestinal tract and cause loss of appetite and dehydration [69]. However, for migrating birds, digestive problems may have severe consequences, by depleting the required body stores for the trip [6], or impairing immune capacity [70].

Despite the previous general perception of very low incidence of haematozoan infections in marine and coastal birds [12,71,72], data provided in recent studies suggest that the incidence of haematozoan infections in marine birds is higher than previously thought (rev. Quillfeldt *et al*. [73]). Our results show an overall haematozoan prevalence of 34% in gulls of the *Larus fuscus* taxonomic group. Infections by avian haematozoa are known to impair several physiological functions and significantly affect flying performance [12,74]. Therefore, it seems likely that migratory culling plays a role in maintaining low prevalence of infections among some of the long-distance migratory populations [14]. However, our results show extensive geographical variation in the prevalence of hemoparasites, not associated to migratory habits.

## Conclusions

Our study explored the links between animal migration and disease risk, and found large differences among populations in parameters of anti-parasite defence and in the prevalence of avian pathogenic diseases. Our results offer partial support for a role of migration in disease dynamics of Lesser black-backed gulls. We found higher activity of constitutive immunity in long-distance migrants, which is in line with previous studies suggesting that long-distance migration selects for higher investment in immunity [15]. In addition, we found higher seroprevalence of one of the most common serotypes of avian influenza in populations with short-distance migratory strategy, supporting the idea that adopting a resident or short-distance migratory strategy may result in higher disease prevalence at the population level [14]. However, we found large geographical variation in other immune parameters and prevalence of other viral and protozoan infections, suggesting important variation in disease dynamics at the population level not explained by differences in migratory strategy.

## Supporting Information

S1 Table Data set used in the study. Delta = Delta Ebro (Spain); Häme = Finland; Kokkola = Finland; Moerdjik = The Netherlands; Sisargas = Galicia (Spain); Agglu = agglutination score; Hp = haptoglobin concentration; Lysozyme = lysozyme activity (OD); H = heterophils; L = lymphocytes; E = eosinophils; B = basophils; M = monocytes; Haemoproteus = infection status; FluA = prevalence of antibodies against influenza A; PMV1–6 = prevalence of antibodies against avian paramixovirus 1, 4 or 6.
(DOCX)
