# Supplementary material for: Variation in Immune Parameters and Disease Prevalence among Lesser Black-Backed Gulls (Larus fuscus sp.) with Different Migratory Strategies
Source: PLoS One. 2015 Feb 13;10(2):e0118279. doi: 10.1371/journal.pone.0118279 (PMC4334556; doi:10.1371/journal.pone.0118279)
Supplement: S1 Table — Delta = Delta Ebro (Spain); Häme = Finland; Kokkola = Finland; Moerdjik = The Netherlands; Sisargas = Galicia (Spain); Agglu = agglutination score; Hp = haptoglobin concentration; Lysozyme = lysozyme activity (OD); H = heterophils; L = lymphocytes; E = eosinophils; B = basophils; M = monocytes; Haemoproteus = infection status; FluA = prevalence of antibodies against influenza A; PMV1–6 = prevalence of antibodies against avian paramixovirus 1, 4 or 6. (DOCX) [file pone.0118279.s001.docx]

Variation in immune parameters and disease prevalence among Lesser Black-backed Gulls (*Larus fuscus* sp.) with different migratory strategies. Arriero et al. 2014

Electronic Supplementary Material

ESM1. Data set used in the study (Delta= Delta Ebro (Spain); Häme= Finland; Kokkola = Finland; Moerdjik= The Netherlands; Sisargas= Galicia (Spain); Agglu= agglutination score; Hp= haptoglobin concentration; Lysozyme= lysozyme activity (OD); H= heterophils; L= lymphocytes; E= eosinophils; B= basophils; M= monocytes; Haemoproteus= infection status; FluA= prevalence of antibodies against influenza A; PMV1-6= prevalence of antibodies against avian paramixovirus 1, 4 or 6.

| SITE | ID | SEX | Lysis | Agglu | Hp | Lysozyme | H | L | E | B | M | Haemoproteus | FluA | PMV1 | PMV4 | PMV6 |
| --- | --- | --- | --- | --- | --- | --- | --- | --- | --- | --- | --- | --- | --- | --- | --- | --- |
| Delta | 6168498 | M | 3 | 4 | 0.051 | 0.994 |  |  |  |  |  | 1 | 1 | 0 | 0 | 1 |
| Delta | 6168500 | F | 3.5 | 4.5 | 0.159 | 1.051 | 25 | 75 | 0 | 0 | 0 | 0 | 1 | 1 | 0 | 0 |
| Delta | 6168483 | F | 3 | 4 | 0.054 | 1.011 | 58 | 42 | 0 | 0 | 0 | 0 | 1 | 0 | 0 | 1 |
| Delta | 6168485 | F | 3 | 4 | 0.052 | 0.986 | 15.2 | 82.6 | 0 | 0 | 2.2 | 0 | 1 | 1 | 0 | 0 |
| Delta | 6118850 | F | 3 | 6 | 0.062 | 1.019 |  |  |  |  |  | 0 | 1 | 1 | 0 | 1 |
| Delta | 6118854 | M | 3 | 8 |  | 0.956 |  |  |  |  |  | 0 | 1 | 1 | 0 | 1 |
| Delta | 6180609 | F | 4 | 7 | 0.165 | 1.088 |  |  |  |  |  | 0 | 1 | 1 | 0 | 1 |
| Delta | 6180611 | F | 3 | 8 | 0.099 | 1.029 |  |  |  |  |  | 0 | 1 | 1 | 0 | 0 |
| Delta | 6180612 | M | 4 | 6 | 0.069 | 1.057 |  |  |  |  |  | 0 | 1 | 0 | 0 | 0 |
| Delta | 6180613 | M | 3 | 7 | 0.110 | 1.054 |  |  |  |  |  | 0 | 1 | 1 | 0 | 1 |
| Delta | 6180617 | F | 3 | 7 | 0.164 | 1.192 |  |  |  |  |  | 0 | 1 | 0 | 0 | 1 |
| Delta | 6180622 | M | 3 | 7 | 0.061 | 1.005 |  |  |  |  |  | 0 | 0 | 0 | 0 | 0 |
| Delta | 6180623 | F | 3 | 6 | 0.076 | 0.995 |  |  |  |  |  | 0 | 1 | 1 | 0 | 1 |
| Delta | 6180625 | F | 3 | 7 | 0.184 | 1.079 |  |  |  |  |  | 0 | 1 | 1 | 0 | 1 |
| Delta | 6180626 | F | 4 | 8 | 0.046 | 1.103 |  |  |  |  |  | 0 | 1 | 1 | 0 | 0 |
| Delta | 6180629 | M | 3 | 7 | 0.018 | 0.996 |  |  |  |  |  | 0 | 1 | 1 | 0 | 0 |
| Delta | 6174930 | M | 3.5 | 6 | 0.150 | 1.027 | 55.4 | 43.6 | 0 | 0 | 1.0 | 0 | 0 | 1 | 0 | 0 |
| Delta | 6174937 | M | 3 | 4 | 0.049 | 1.023 | 29 | 69 | 0 | 0 | 2 | 0 | 0 | 1 | 0 | 0 |
| Delta | 6174938 | F | 3 | 5 | 0.042 | 1.119 | 34 | 66 | 0 | 0 | 0 | 0 | 1 | 0 | 0 | 0 |
| Delta | 6174941 | F | 2.5 | 4.5 | 0.053 | 1.034 |  |  |  |  |  | 1 | 0 | 1 | 0 | 0 |
| Delta | 6174945 | M | 3 | 5 | 0.084 | 1.008 | 16 | 83 | 0 | 0 | 1 | 1 | 1 | 1 | 0 | 1 |
| Delta | 6174946 | F | 2 | 3.5 | 0.181 | 1.096 | 43 | 57 | 0 | 0 | 0 | 0 | 1 | 0 | 0 | 0 |
| Delta | 6174947 | F | 4 | 6 | 0.095 | 1.061 | 46.3 | 53.7 | 0 | 0 | 0 | 0 | 1 | 1 | 0 | 1 |
| Delta | 6174950 | F | 2 | 3 | 0.052 | 1.009 | 73.3 | 26.7 | 0 | 0 | 0 | 1 | 0 | 1 | 0 | 1 |
| Delta | 6135190 | F | 3 | 4.5 | 0.054 | 0.966 |  |  |  |  |  | 0 | 1 | 1 | 0 | 1 |
| Delta | 6180601 | F | 3 | 5 | 0.058 | 1.004 | 28 | 72 | 0 | 0 | 0 | 1 | 1 | 0 | 0 | 0 |
| Delta | 6180602 | F | 3 | 5 | 0.047 | 0.982 |  |  |  |  |  | 0 | 1 | 1 | 0 | 0 |
| Delta | 6180603 | F | 3 | 4 | 0.045 | 1.028 | 41.4 | 58.6 | 0 | 0 | 0 | 0 | 0 | 1 | 0 | 0 |
| Delta | 6180607 | F | 2.5 | 5 | 0.046 | 1.039 | 64 | 35 | 0 | 0 | 1 | 0 | 1 | 1 | 0 | 1 |
| Delta | 6180608 | F | 3 | 4 | 0.057 | 0.944 |  |  |  |  |  | 0 | 1 | 0 | 0 | 1 |
| Hame | CT154195 | F | 3 | 7 | 0.058 | 0.975 |  |  |  |  |  | 0 | 1 | 1 | 0 | 1 |
| Kokkola | HT254615 | M | 3 | 5 | 0.112 | 1.157 | 33 | 67 | 0 | 0 | 0 | 0 | 1 | 1 | 0 | 1 |
| Hame | CT154194 | M | 3 | 6 | 0.068 | 0.935 | 41 | 59 | 0 | 0 | 0 | 0 | 0 | 0 | 0 | 1 |
| Hame | HT201100 | F | 3 | 7 | 0.050 | 0.998 | 29 | 69 | 0 | 0 | 2 | 0 | 1 | 0 | 0 | 0 |
| Hame | HT182429 | M | 7 | 9 | 0.550 | 1.110 | 46 | 54 | 0 | 0 | 0 | 0 | 0 | 1 | 0 | 0 |
| Hame | CT154196 | M | 9 | 10 | 0.130 | 1.264 | 33 | 67 | 0 | 0 | 0 | 0 | 1 | 0 | 0 | 0 |
| Hame | HT233286 | M | 4 | 6.5 | 0.111 | 1.001 | 37 | 63 | 0 | 0 | 0 | 0 | 1 | 0 | 0 | 0 |
| Hame | HT244499 | M | 3 | 6 | 0.052 | 1.059 |  |  |  |  |  | 0 | 0 | 0 | 0 | 1 |
| Hame | HT193999 | F | 3 | 5 | -0.028 | 1.072 | 24 | 75 | 0 | 0 | 1 | 0 | 0 | 1 | 0 | 0 |
| Hame | CT154193 | F | 2 | 6 | 0.250 | 1.151 | 50 | 50 | 0 | 0 | 0 | 1 | 0 | 0 | 0 | 0 |
| Hame | HT179893 | M | 3 | 6 | -0.015 | 1.052 | 33 | 65 | 0 | 0 | 2 | 1 | 0 | 0 | 0 | 1 |
| Hame | HT211260 | M | 2 | 7 | 0.040 | 1.010 | 52 | 48 | 0 | 0 | 0 | 1 | 0 | 0 | 0 | 0 |
| Hame | CT154163 | F | 3 | 7 | 0.050 | 1.028 | 55 | 43 | 0 | 0 | 2 | 0 | 1 | 0 | 0 | 1 |
| Kokkola | CT154187 | F | 3 | 6 | 0.060 | 1.039 | 41 | 56 | 0 | 0 | 3 | 0 | 1 | 0 | 0 | 1 |
| Kokkola | CT154188 | M | 1 | 5 | 0.090 | 0.958 | 66 | 33 | 0 | 0 | 1 | 0 | 0 | 1 | 0 | 0 |
| Kokkola | CT154189 | M | 4 | 7 | 0.023 | 0.961 | 54.0 | 46.0 | 0 | 0 | 0 | 0 | 0 | 0 | 0 | 0 |
| Kokkola | CT154190 | F | 1 | 6 | 0.183 | 0.976 | 42.9 | 57.1 | 0 | 0 | 0 | 0 | 1 |  |  |  |
| Kokkola | CT154191 | M | 3 | 6 | 0.294 | 1.021 | 26 | 71 | 0 | 0 | 3 | 0 | 1 | 0 | 0 | 1 |
| Kokkola | CT154192 | F | 3 | 6 | 0.076 | 0.998 | 55 | 45 | 0 | 0 | 0 | 0 | 1 | 1 | 0 | 1 |
| Hame | CT154196 | F | 2 | 6 | -0.020 | 0.998 | 37 | 61 | 2 | 0 | 0 | 0 | 1 | 0 | 0 | 0 |
| Hame | CT154198 | F | 3 | 6 | 0.063 | 1.161 | 46 | 54 | 0 | 0 | 0 | 0 | 1 | 1 | 0 | 0 |
| Hame | CT154199 | F | 3 | 6 | 0.052 | 0.976 | 27.8 | 72.2 | 0 | 0 | 0 | 1 | 0 | 1 | 0 | 1 |
| Hame | CT154200 | M | 3 | 8 | 0.095 | 1.125 | 72 | 27 | 0 | 0 | 1 | 1 | 0 | 1 | 0 | 1 |
| Moerdjik | 5460631 | M | 2 | 4 | 0.145 | 0.926 | 33.3 | 66.7 | 0 | 0 | 0 | 0 | 0 | 0 | 0 | 0 |
| Moerdjik | 5460632 | M | 2.5 | 4 | 0.300 | 0.922 |  |  |  |  |  | 0 | 0 | 0 | 0 | 0 |
| Moerdjik | 5460633 | F | 2 | 5 | 0.297 | 0.961 | 52 | 46 | 0 | 0 | 2 | 0 | 1 |  |  |  |
| Moerdjik | 5460634 | F | 2.5 | 4.5 | 0.269 | 0.980 | 30 | 70 | 0 | 0 | 0 | 0 | 1 | 0 | 0 | 0 |
| Moerdjik | 5460635 | M | 2.5 | 5 | 0.184 | 1.008 | 68.8 | 31.3 | 0 | 0 | 0 | 0 | 1 | 0 | 0 | 0 |
| Moerdjik | 5460636 | M | 3 | 5 | 0.261 | 1.001 | 27 | 73 | 0 | 0 | 0 | 0 | 0 | 0 | 0 | 0 |
| Moerdjik | 5460630 | M | 3 | 4 | 0.052 | 0.977 | 26 | 73 | 0 | 0 | 1 | 1 | 0 | 1 | 0 | 0 |
| Moerdjik | 5460637 | F | 3 | 5 | 0.113 | 1.041 | 27 | 72 | 0 | 0 | 1 | 1 | 1 | 1 | 0 | 0 |
| Moerdjik | 5460659 | M | 3.5 | 5 | 0.067 | 1.019 | 40 | 57 | 0 | 0 | 3 | 1 | 0 | 0 | 0 | 1 |
| Moerdjik | 5460660 | M | 3 | 5 | 0.098 | 0.966 | 35 | 64 | 0 | 0 | 1 | 1 | 1 | 1 | 0 | 0 |
| Moerdjik | 5460661 | F | 3 | 4.5 |  |  | 25 | 74 | 0 | 0 | 1 | 0 |  |  |  |  |
| Moerdjik | 5460638 | M | 3 | 4 | 0.086 | 1.266 | 47 | 51 | 0 | 0 | 2 | 1 | 1 | 1 | 0 | 0 |
| Moerdjik | 5460639 | M | 2 | 5 | 0.107 | 1.062 | 21 | 78 | 0 | 0 | 1 | 0 | 0 | 0 | 0 | 0 |
| Moerdjik | 5460640 | M | 3 | 5 | 0.277 | 1.004 | 57 | 43 | 0 | 0 | 0 | 1 | 1 |  |  |  |
| Moerdjik | 5460641 | M |  |  |  |  | 38 | 62 | 0 | 0 | 0 | 0 |  |  |  |  |
| Moerdjik | 5460644 | M | 2.5 | 4 | 0.146 | 0.996 | 25 | 75 | 0 | 0 | 0 | 0 | 1 | 0 | 0 | 0 |
| Moerdjik | 5460647 | M | 2 | 3 | 0.088 | 0.969 | 35 | 64 | 0 | 0 | 1 | 1 | 0 | 0 | 0 | 0 |
| Moerdjik | 5460650 | F | 2 | 4 | 0.051 | 1.082 | 25 | 74 | 0 | 0 | 1 | 1 | 1 | 0 | 0 | 0 |
| Moerdjik | 5460651 | F | 3.5 | 6 | 0.104 | 1.413 | 53 | 46 | 0 | 0 | 1 | 0 | 1 | 0 | 0 | 0 |
| Moerdjik | 5460652 | M | 3.5 | 3.5 | 0.053 | 0.988 | 20 | 80 | 0 | 0 | 0 | 1 | 1 | 0 | 0 | 0 |
| Moerdjik | 5460653 | F | 4 | 6 | 0.732 | 1.067 | 33 | 63 | 0 | 0 | 4 | 0 | 1 | 0 | 0 | 0 |
| Moerdjik | 5460654 | F |  |  |  |  | 65 | 35 | 0 | 0 | 0 | 0 |  |  |  |  |
| Moerdjik | 5460655 | F | 3 | 7 | 0.143 | 1.020 | 49 | 51 | 0 | 0 | 0 | 0 | 0 | 1 | 0 | 0 |
| Moerdjik | 5460656 | F |  |  |  |  | 15 | 84 | 0 | 0 | 1 | 1 |  |  |  |  |
| Moerdjik | 5366859 | F | 3 | 4 | 0.064 | 1.019 | 32 | 67 | 0 | 0 | 1 | 1 | 1 | 0 | 0 | 0 |
| Moerdjik | 5460657 | M | 3 | 4.5 | 0.058 | 1.016 | 43 | 53 | 0 | 1 | 3 | 1 | 0 | 0 | 0 | 0 |
| Moerdjik | 5460658 | F | 3 | 7 | 0.065 | 1.043 | 16 | 84 | 0 | 0 | 0 | 0 | 1 | 0 | 0 | 0 |
| Moerdjik | 5410830 | F | 2 | 5 | 0.610 |  | 33 | 67 | 0 | 0 | 0 | 0 |  |  |  |  |
| Sisargas | 6188351 | F | 3 | 4 | 0.085 | 1.092 | 39 | 60 | 0 | 0 | 1 | 0 | 1 | 1 | 0 | 1 |
| Sisargas | 6188352 | F | 4 | 7 | 0.078 | 1.078 | 64 | 36 | 0 | 0 | 0 | 1 | 1 | 0 | 0 | 0 |
| Sisargas | 6188354 | F | 3 | 5 | 0.188 | 1.048 | 41 | 55 | 0 | 0 | 4 | 1 | 1 | 1 | 0 | 0 |
| Sisargas | 6188353 | F | 4 | 5 | 0.098 | 1.062 | 44 | 54 | 0 | 0 | 2 | 0 | 1 | 1 | 0 | 0 |
| Sisargas | 6188355 | F | 3 | 6 | 0.068 | 1.432 | 23 | 77 | 0 | 0 | 0 | 0 | 1 | 0 | 0 | 0 |
| Sisargas | 6188356 | F | 3 | 5 | 0.046 | 1.073 | 17 | 80 | 0 | 0 | 3 | 1 | 1 | 0 | 0 | 0 |
| Sisargas | 6188357 | F | 3 | 4 | 0.040 | 1.781 | 34 | 65 | 0 | 0 | 1 | 0 | 0 | 1 | 0 | 0 |
| Sisargas | 6188358 | F | 3 | 3 | 0.076 | 1.488 | 41.6 | 54.5 | 0 | 0 | 4.0 | 1 | 1 | 1 | 0 | 0 |
| Sisargas | 6188359 | M | 3 | 4 | 0.065 | 1.029 |  |  |  |  |  | 1 | 1 | 0 | 0 | 0 |
| Sisargas | 6188360 | F | 3 | 3 | 0.072 | 1.080 | 61 | 39 | 0 | 0 | 0 | 1 | 0 | 0 | 0 | 0 |
| Sisargas | 6188361 | M | 3 | 6 | 0.078 | 1.077 | 11 | 89 | 0 | 0 | 0 | 0 | 1 | 0 | 0 | 0 |
| Sisargas | 6188362 | M | 4 | 6 | 0.044 | 0.998 | 53 | 45 | 0 | 0 | 2 | 0 | 1 | 1 | 0 | 0 |
| Sisargas | 6188363 | F | 3.5 | 4 | 0.094 | 1.046 | 58.7 | 38.1 | 0 | 0 | 3.2 | 1 | 1 | 0 | 0 | 0 |
| Sisargas | 6188364 | M | 3 | 4 | 0.277 | 0.935 | 29 | 69 | 0 | 0 | 2 | 1 | 1 |  |  |  |
| Sisargas | 6188365 | M | 3 | 5 | 0.037 | 1.019 | 49 | 50 | 0 | 0 | 1 | 1 | 1 | 1 | 0 | 0 |
| Sisargas | 6188366 | F | 3 | 5 | -0.024 | 1.091 | 42.1 | 56.1 | 0 | 0 | 1.8 | 1 | 1 | 0 | 0 | 1 |
| Sisargas | 6188367 | F | 3 | 4 | -0.016 | 1.073 | 47 | 53 | 0 | 0 | 0 | 1 | 1 | 0 | 0 | 0 |
| Sisargas | 6188368 | M | 2 | 3 | 0.091 | 1.012 | 76 | 23 | 0 | 0 | 1 | 1 | 1 | 0 | 0 | 0 |
| Sisargas | 6188369 | F | 3.5 | 4 | 0.059 | 1.084 | 42.1 | 55.3 | 0 | 0 | 2.6 | 0 | 1 | 1 | 0 | 0 |
| Sisargas | 6188370 | M | 3.5 | 5 | 0.077 | 1.004 | 62 | 37 | 0 | 0 | 1 | 0 | 1 | 1 | 0 | 0 |
| Sisargas | 6188371 | M | 4 | 5 | 0.125 | 1.070 | 38 | 61 | 0 | 0 | 1 | 0 | 1 | 0 | 0 | 0 |
| SPN | 6188372 | M | 3 | 4 | 0.016 | 1.020 | 7 | 93 | 0 | 0 | 0 | 0 | 1 | 0 | 0 | 0 |
| SPN | 6188373 | M | 3 | 4 | 0.157 | 1.014 | 73.3 | 26.7 | 0 | 0 | 0 | 1 | 0 | 0 | 0 | 0 |
| SPN | 6188374 | M | 3 | 4 | 0.564 | 1.005 | 51 | 47 | 0 | 0 | 2 | 0 | 1 | 0 | 0 | 0 |
| SPN | 6188375 | M | 3.5 | 4 | 0.130 | 1.037 | 65.3 | 33.3 | 0 | 0 | 1.3 | 1 | 1 | 0 | 0 | 0 |
